# Network Pharmacology Approach to Investigate the Mechanism of Danggui-Shaoyao-San against Diabetic Kidney Disease

**DOI:** 10.1155/2023/9208017

**Published:** 2023-01-03

**Authors:** Yulian Chen, Xiaodan Song, Yunxia Luo, Guandong Li, Yueming Luo, Ziyan Wang, Riming He, Jiandong Lu, Guoliang Xiong, Hong Cheng, Huilin Li, Shudong Yang

**Affiliations:** ^1^Shenzhen Traditional Chinese Medicine Hospital, Guangzhou University of Chinese Medicine, Shenzhen, Guangdong, China; ^2^The Second Affiliated Hospital, Guangzhou University of Chinese Medicine, Guangzhou, Guangdong, China; ^3^School of Traditional Chinese Medicine, Guangdong Pharmaceutical University, Guangzhou, Guangdong, China; ^4^Nanchang Medical College, Nanchang, China; ^5^Shenzhen Hospital of Guangzhou University of Chinese Medicine (FuTian), Shenzhen, Guangdong, China

## Abstract

**Background:**

Danggui-Shaoyao-San (DSS) is a traditional Chinese medicine formula that has been widely used to treat a variety of disorders, including renal diseases. Despite being well-established in clinical practice, the mechanisms behind the therapeutic effects of DSS on diabetic nephropathy (DN) remain elusive.

**Methods:**

To explore the therapeutic mechanism, we explored the action mechanism of DSS on DN using network pharmacology strategies. All ingredients were selected from the relevant databases, and active ingredients were chosen on the basis of their oral bioavailability prediction and drug-likeness evaluation. The putative proteins of DSS were obtained from the Traditional Chinese Medicine Systems Pharmacology (TCMSP) database, whereas the potential genes of DN were obtained from the GeneCards and OMIM databases. Enrichment analysis using gene ontology (GO) and the Kyoto encyclopedia of genes and genomes (KEGG) was performed to discover possible hub targets and gene-related pathways. Afterwards, the underlying molecular mechanisms of DSS against DN were validated experimentally in vivo against db/db mice.

**Results:**

We identified 91 phytochemicals using the comprehensive network pharmacology technique, 51 of which were chosen as bioactive components. There were 40 proteins and 20 pathways in the target-pathway network. The experimental validation results demonstrated that DSS may reduce the expression of TNF-*α*, IL-6, and ICAM-1, as well as extracellular matrix deposition, by blocking the JNK pathway activation, which protects against kidney injury.

**Conclusion:**

This study discovered the putative molecular mechanisms of action of DSS against diabetic kidney damage through a network pharmacology approach and experimental validation.

## 1. Introduction

The increasing prevalence of diabetic nephropathy (DN) parallels the dramatic rise in the prevalence of diabetes globally. Nearly half of all type 2 diabetic (T2D) patients and one-third of type 1 diabetic (T1D) patients eventually suffer from CKD [1]. Every year, around 25 out of every 10,000 diabetic patients are diagnosed with end-stage renal disease [2]. Diabetic nephropathy etiology is complicated, and the treatment methods are limited and only delay disease progression [3]. Currently, the use of conventional approaches, including aldosterone system blockage, blood glucose level management, and bodyweight reduction, does not always produce satisfying results, as they are not effective for preventing diabetes from progressing to end-stage renal disease [4, 5]. As a result, it is necessary to develop more effective therapies for DN patients. TCM could be an alternative to western medicine for treating DN. For example, Chinese herbal medicine and acupuncture reportedly have therapeutic benefits for DN [6].

TCM places emphasis on the notion of the integrity of the entire human body. Different from the single-target curative effect of chemical drugs, TCM compound ingredients have an overall curative effect, which is usually regulated by “multicomponent” and “multitarget” [7]. Network pharmacology is efficient in building a “compound-protein/gene-disease” network, which is able to describe complexities among biological systems, medicines, and illnesses from a network viewpoint and has a holistic philosophy similar to TCM [8]. By providing the compound-target and target-pathway networks, network pharmacology aids in evaluating the rationale and compatibility of TCM [9]. In conclusion, network pharmacology is a rational application for drug discovery, notably in the area of TCM preparations' studies and development [10, 11]. Danggui-Shaoyao-San (DSS) is a formula made up of six Chinese herbs, including Paeoniae Radix Alba (PRA), Angelica Sinensis Radix (ASR), Chuanxiong Rhizoma (CR), Poria cocos (PC), Atractylodis Macrocephalae Rhizoma (AMR), and Alismatis Rhizoma (AR), which has long been employed as a blood-quickeningstasis-transforming formula for gynecological disorders in China, such as algomenorrhea, irregular menstruation, menopausal syndrome, and infertility [12–15]. Recent studies have mainly focused on therapeutic efficacy for neural dysfunctions, including depression, Alzheimer's disease, poor memory, and other neuropsychiatric symptoms [16–19]. But emerging evidence has also revealed the therapeutic efficacy of drugs for renal disease, including fibrosis [20], diabetic nephropathy [21, 22], nephrotic syndrome [23], and so on. Despite the fact that so many favorable benefits have been demonstrated, the underlying mechanisms of DSS in DN have not been previously explored.

**Figure 1:**
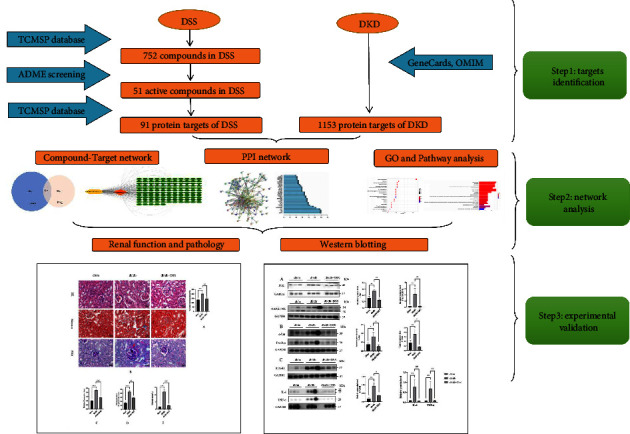
The technical summary of the current study.

In this current study, we adopted a network pharmacology approach to analyze the underlying mechanisms of DSS in DN. Furthermore, the potential targets of DSS against DN hypothesized by the network pharmacological approach were confirmed through in vivo experiments. The detailed graphical abstract of this work is illustrated in Figure 1.

## 2. Materials and Methods

### 2.1. Network Pharmacology-Based Analysis

#### 2.1.1. Screening of Active Components in DSS

Active DSS compounds were retrieved from the TCM systems pharmacology database and analysis platform (TCMSP, http://tcmspw.com), the encyclopedia of TCM (ETCM), and the TCM integrative pharmacology-based research platform (TCMIP), which lists the pharmacokinetic properties of active phytochemicals such as oral bioavailability (OB) and drug-likeness (DL), among others. The OB index is an important pharmacokinetic parameter that represents the proportion of the medication in the blood circulation. The similarity between a novel compound and a pre-existing drug can be expressed in terms of the DL index; a high DL indicates that although a compound is not yet used as a drug, it may become one in the future. In this study, the DSS bioactive components meriting further analysis were those with an OB ≥ 30% and DL ≥ 0.18.

#### 2.1.2. Disease-Targets-Compound Network Construction and Analysis

Potential targets of DSS were predicted using the TCMSP, while target genes for diabetic nephropathy were collected from the GeneCards and OMIM databases. Subsequently, we used the target genes of the active DSS components and DN therapeutic targets obtained from the databases to identify overlapping genes. We applied Cytoscape 3.8.0 software to construct the “disease-targets-compound network” visual network diagram.

#### 2.1.3. PPI Network Construction

Targets common to both diabetic nephropathy and DSS were imported into the STRING database, with the research species set as human. Protein relationships were then obtained; those with values >0.4 were screened, and the free ends were removed. Finally, a PPI protein interaction network diagram was drawn; more adjacent genes in the PPI map play a more important role.

#### 2.1.4. Gene Ontology and Pathway Enrichment Analysis

GO and Kyoto encyclopedia of genes and genomes (KEGG) enrichment analyses were performed using the DAVID 6.8 functional annotation tool (https://david.ncifcrf.gov/). A false discovery rate cut-off <0.05 was applied for clustering during the functional annotation.

## 3. Experimental Validation

### 3.1. Preparation of the DSS Extract

DSS comprises ASR, PRA, CR, PC, AMR, and AR (a ratio of 3 : 16 : 8 : 8 : 4 : 4). It was provided by the pharmacy department of the Shenzhen Traditional Chinese Medicine Hospital and prepared in the following manner: the mixture is submerged in distilled water (1 : 8, *w*/*v*) at room temperature for 30 minutes with a reflux device before boiling for 1 hour and filtering the extract. The boiling and extraction steps were performed two times. The extracted filtrate was combined and then concentrated to a final extract concentration of 0.64 g/ml, referred to as DSS extract. The extract was dried to a powder form by freeze-drying and stored at −20°C, and the powder was redissolved with Milli-Q water to obtain DSS extract before the treatment.

### 3.2. Animals and Treatment

Male diabetic mice (db/db) and mice (db/m) of 10 weeks were purchased from Jiangsu Jicui Yaokang Biotechnology Co., Ltd. (Nanjing, China). The mice were maintained in the specific pathogen-free animal room of Shenzhen Top Biotech Co., Ltd. (Shenzhen, China) on a 12-h light/dark cycle with a relative humidity of 40–60% and temperature of 20–25°C. Animals were free to access food and water. Fasting blood glucose greater than 11.1 mmol/L after 30 days was diagnosed as diabetes. The diabetic mice were randomly assigned to the db/db group and the DSS group (*n* = 5). Eight db/m mice were used as a control group (*n* = 8). The db/db group and DSS group received gavages of 0.9% saline (10 ml/kg/day) and the DSS extract (6.4 g/kg/day), respectively. The study was approved by the Animal Ethics Committee of Shenzhen Top Biotech Co., Ltd. and conducted in full compliance with the directives of the National Institutes of Health guidelines. All mice were killed through cervical dislocation without consciousness at the end of the study. Blood samples were collected by eye enucleation, and the kidneys were excised and promptly snap-frozen to detect various markers.

### 3.3. Serum Biochemical Analysis

Samples of serum were taken and centrifuged for 15 minutes at 1,500 rpm in sterile tubes. A serum creatinine (Scr) kit was used to assess the Scr levels according to the manufacturer's instructions.

### 3.4. Histological Examination

3-*μ*m sections were prepared from the kidneys after dehydration, embedment in wax, and slicing for the periodic-acid-schiff (PAS), Masson's trichrome staining (Masson), and hematoxylin and eosin staining (H&E). Images were visualized with a Zeiss optical microscope. Ninety glomeruli from three mice in each group were assessed for the degree of glomerular mesangial expansion and glomerular area employing Image-J (NIH, Bethesda, MD, USA) in a blinded fashion, under ×400 magnification by PAS staining. Quantification of fibrosis was further performed under ×200 magnification by Masson trichrome staining [24].

### 3.5. Western Blotting

In brief, proteins extracted from the kidneys were lysed with RIPA buffer (Sigma-Aldrich, USA). The protein concentration was detected by the BCA Protein Assay Kit (UB276926, Thermo Fisher Scientific, USA). Prepared protein lysates were loaded onto 8–12% SDS-PAGE gels and electrophoretically transferred to a polyvinylidene fluoride membrane. After being blocked with 5% nonfat milk for 1 h at room temperature, the membranes were probed with primary antibodies, including anti-JNK1 + JNK2 + JNK3 (ab124956, Abcam, 1 : 1,000), anti-c-Jun (ab40766, Abcam, 1 : 1,1000), Phosphoa-c-Jun (ab32385, Abcam, 1 : 1,1000), anti-ICAM-1 (ab171123, Abcam, 1 : 1,000), TNF-*α* (sc-52746, Santa Cruz Biotechnology, 1 : 1,000), IL-6 (ab9324, Abcam, 1 : 1,000), Phospho-SAPK/JNK (#4668, Cell Signaling Technology, 1 : 1,000), and GAPDH (#2118, Cell Signaling Technology, 1 : 1000). And then the blots were incubated with horseradish peroxidase-conjugated secondary antibodies (ab6789, ab6721, Abcam, 1 : 2,000) for 1 h at room temperature. The specific reaction was imaged with an advanced ECL kit (GE Healthcare, UK) and the ChemiDoc MP Imaging System (Bio-Rad Laboratories, USA). ImageJ software was employed for the densitometric study.

## 4. Statistical Analysis

The data were expressed as means ± SEM. SPSS (23.0; SPSS Inc., USA) was employed for analyses. One-way analysis of variance was used to detect differences between groups, followed by Tukey's multiple comparisons test. A *P* value <0.05 was considered significant.

## 5. Result

### 5.1. Active Components in DSS

A total of 752 ingredients were identified through the TCMSP database, ETCM, and TCMIP v2.0 (up to July 2022). Based on the cut-offs of OB ≥ 30% and DL ≥ 0.18 detailed above, 51 candidate bioactive components were subjected to further analyses after eliminating overlapping genes. Detailed information is shown in Table 1.

### 5.2. Disease-Targets-Compound Network Construction and Analysis

**Figure 2:**
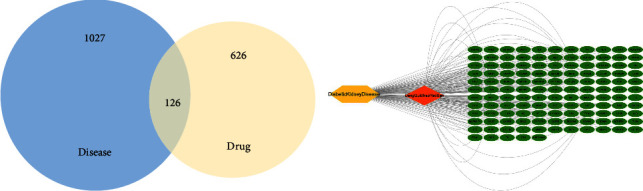
(a) The 126 overlapping genes between diabetic kidney disease (1027 genes) and DSS (626 genes). (b) The disease-targets-compound network for DSS on diabetic kidney disease (nodes with different colors and forms indicate different types of information in the figure. Common targets are represented by green ovals, DKD by yellow hexagons, and DSS by orange prisms).

Ninety-one potential DSS targets were predicted by the TCMSP database. A total of 1,153 targets related to diabetic nephropathy were collected from the GeneCards and OMIM databases. After eliminating the overlaps, we identified 126 common (i.e., both drug and disease target genes) (Figure 2(a)), suggesting that these genes potentially play a significant role in DSS treatment of DN. To better understand the potential mechanism of DSS in DN, we constructed a disease-targets-compound network using Cytoscape 3.8.0 software (Figure 2(b)). Nodes with different colors and shapes in the figure represent different types of information. Green ovals represent common targets, blue rectangular nodes represent active ingredients of TCM, purple hexagons represent DN, and orange prisms represent DSS.

### 5.3. Target Proteins PPI Network Construction and Analysis

**Figure 3:**
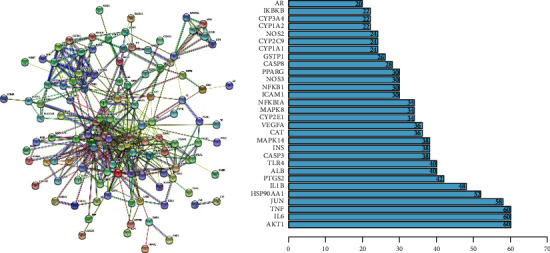
(a) The PPI network of targets for both diabetic nephropathy and DSS is obtained from the STRING database. (b) The bar plot of the top 30 hub genes in the PPI network.

To determine how the overlapping genes interact, the 126 common targets were imported into the STRING database, and a PPI network diagram was drawn (Figure 3(a)). As stated above, more adjacent genes in the PPI map played more important roles. By calculating the number of nodes connected to each gene, the top 30 genes in terms of centrality were identified as the most important genes of DSS with respect to the treatment of CKD (Figure 3(b)); these included IL-6, AKT1, TNF, CAT, JUN, and PTGS2.

### 5.4. Gene Ontology and KEGG Enrichment Analyses

**Figure 4:**
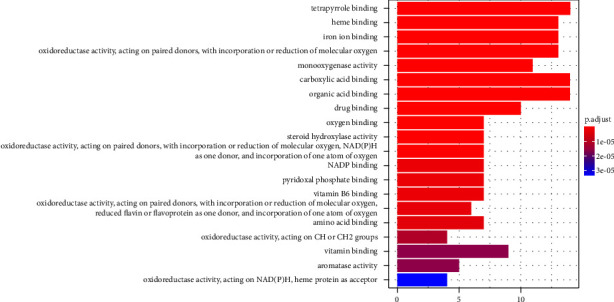
The top 20 significantly enriched molecular function from GO-based functional enrichment analysis.

We carried out a GO enrichment analysis using the DAVID database to identify the associations of the 126 common genes with DN; 69 GO terms were obtained, of which the top 20 were significantly enriched in terms of MFs (Figure 4). These terms mainly involved heme oxygenase, tetrapyrrole binding, cysteine-type endopeptidase activity during apoptosis, cysteine-type endopeptidase activity in the apoptosis signaling pathway, and peroxidase activity. These results show that DSS improves diabetic nephropathy by regulating various BPs.

**Figure 5:**
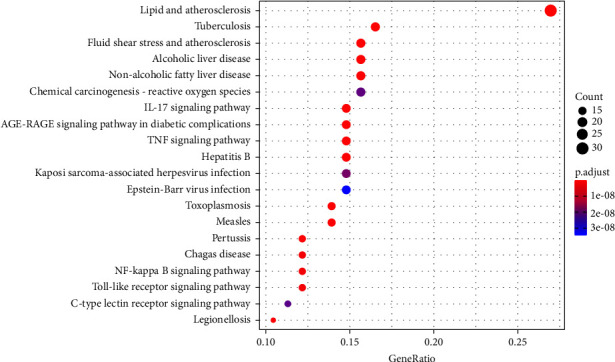
The top 20 significantly enriched pathways from KEGG pathway enrichment analysis.

To further explore how DSS affected DN through the 126 common genes, they were upregulated in the DAVID database and a KEGG enrichment analysis was performed. We identified 140 signaling pathways; the top 20 items are listed in Figure 5 and they include lipid, atherosclerosis, tumor necrosis factor, Posey's sarcoma-related herpes virus infection, Epstein-Barr virus infection, and advanced glycation end product-receptor for AGE (AGE-RAGE) signaling pathways, indicating that the active ingredients in DSS exert their effects through multiple pathways.

### 5.5. DSS Ameliorated Diabetic Kidney Injury in the db/db Mice

**Figure 6:**
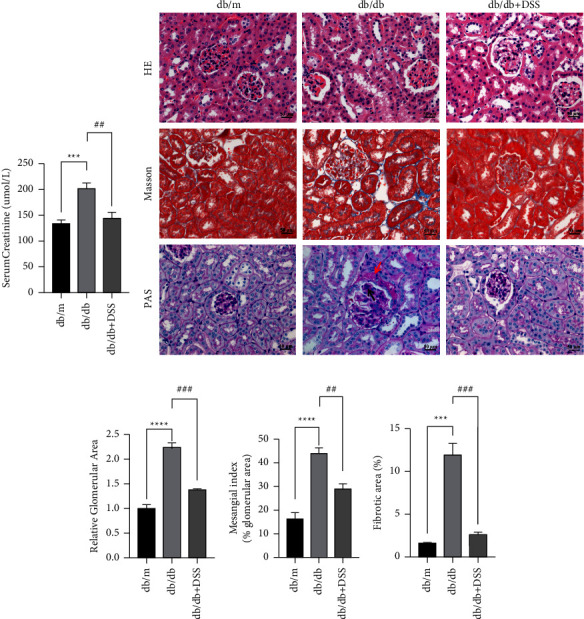
The effect of DSS treatment on diabetic renal functional and pathological injury in the db/db mice. (a) The comparison of the levels of Scr among the three groups. *n* = 5 mice per group. (b) HE, PAS, and Masson staining were used to describe the renal characteristics of different groups. The red deposition represented by the red arrow depicts the evident thickening of the basement membrane of the renal capsule as well as the level of pericystic fibrosis. The Kimmelstiel Wilson nodule is represented by the red deposition shown by the black arrow. *n* = 3 mice per group (×400; scale bar = 50 *μ*m). ((c)–(e)) The relative glomerular area, mesangial index, and interstitial fibrosis score were quantified in the three groups of mice, respectively. *n* = 3 mice per group (^*∗∗∗*^*P* < 0.001, ^*∗∗∗∗*^*P* < 0.0001 vs. the db/m group; ^##^*P* < 0.01, ^###^*P* < 0.001 vs. the db/db group).

Renal function was assessed by the Scr level. The Scr levels in the db/db group increased significantly compared to those in the control group (*P* < 0.01). Administrating DSS significantly reduced the Scr (*P* < 0.01) level in db/db mice (Figure 6(a)). These data indicate that DSS prevented renal functional decline in db/db mice.

The histological pattern of DN includes thickening of the glomerular basement membrane (GBM), mesangial matrix enlargement, nodular glomerulosclerosis, and arteriolar hyalinosis [2]. On PAS staining, db/db mice kidneys exhibited obvious signs of DN. When compared to db/m mice, db/db mice displayed significant glomerular hypertrophy and mesangial matrix enlargement, but these histological alterations were significantly alleviated in DSS-treated mice. Total glomerular area was likewise increased in db/db mice compared to db/m mice, and DSS dramatically lowered this parameter (Figures 6(b)–6(d)). Masson's trichrome staining revealed patchy collagen deposition in the tubular interstitium of the db/db mice, while it was attenuated in DSS-treated db/db mice (Figures 6(b) and 6(f)). Inflammation was severe in these mice. H&E-stained histological lesions were nearly absent in the kidneys of DSS-treated mice (Figure 6(b)). These results indicate that DSS ameliorated renal injury in the db/db mice.

### 5.6. DSS Might Depress Inflammatory Response and Trigger the Inflammatory-Mediated JNK Pathway in the Kidneys of the db/db Mice

**Figure 7:**
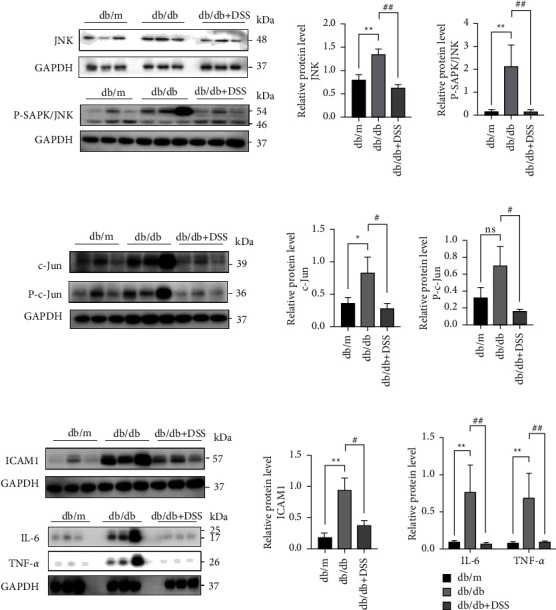
DSS inhibited JNK-mediated inflammatory marker expression in the kidneys of the db/db mice. Representative western blot images and densitometric analysis of JNK and P-SAPK/JNK (a), c-Jun and P-c-Jun (b), ICAM-1, IL-6, and TNF-*α* (c) protein expressions, respectively. Densitometric analysis normalized to GAPDH content. *n* = 3 mice per group (^*∗*^*P* < 0.05, ^*∗∗*^*P* < 0.01vs. the db/m group; #*P* < 0.05,##*P* < 0.01 vs. the db/db group; NS no significance).

The western blot analysis has demonstrated that JNK and c-Jun were upregulated in the db/db mice expression compared with the db/m mice while these proteins were significantly decreased in the DSS group. We also found that the treatment with DSS significantly reduced the phosphorylation levels of JNK and c-Jun in db/db mice (Figures 7(a) and 7(b)). Meanwhile, the inflammatory cytokines, IL-6, ICAM-1, and TNF-*α* were increased in the db/db mice but decreased after the DSS treatment (Figure 7(c)). These data indicated that DSS inhibited the inflammatory response and triggered the JNK pathway in the db/db mice.

## 6. Discussion

Diabetic nephropathy is a multifactorial, complex disease process caused mainly by hyperglycemia, oxidative stress, AGEs, and angiotensin II. All of these factors are linked to various proteins or pathways throughout the development and progression [25]. In particular, Chinese medicine has promised to be the main or alternative treatment for DN due to its multiple targets and functions. Research has focused on discovering the bioactive components and molecular mechanisms for the renoprotective effects of Chinese medicines [26]. TCM formulae are extensively used as the main or supplementary treatment for diabetes mellitus and diabetic nephropathy and have shown promising results in clinical trials [27]. Based on the system biology and multipharmacology, network pharmacology provides a new network model of “multiple targets, multiple effects, and complex diseases,” which is suitable for mechanistic investigation of complex TCM formulae [28]. He et al. found that the AGE-RAGE signaling pathway, the TNF signaling pathway, and the NF-kappa B signaling pathway were critical nodes for the LiuWei DiHuang Pill against type 2 diabetes mellitus (T2DM) throughout the network analysis [29]. This technique was applied in the current investigation to determine the pharmacological mechanism by which DSS alleviates DN.

In this study, the compounds in DSS with an OB of 30% and DL > 0.18 were considered pharmacokinetically active and may be largely responsible for the therapeutic effects of DSS in DN. Paeoniflorgenone was the most abundant compound, followed by paeoniflorin, lactiflorin, paeoniflorin qt, albiflorin qt, benzoyl, paeoniflorin, beta-sitosterol, and stigmasterol, which is consistent with the results of Luo et al. [18].

We demonstrated that DSS acted on several targets and signaling pathways. Hub targets of the pharmacokinetically active compounds included IL-6, AKT1, TNF, CAT, JUN, and PTGS2. Inflammation, cell proliferation, apoptosis, and fibrosis were all linked to these genes. Thus, DSS may exert its antirenal damaging effects in DN by preventing fibrosis, lowering inflammation, and modulating mitochondrial homeostasis, cell proliferation, and apoptosis, which are key mechanisms in the development of DN [30]. Among the pathophysiological mechanisms responsible for the development of DN and the pathogenesis of diabetic neuropathy, inflammation is crucial and has become an important target in DN therapy [31]. DSS may exert therapeutic effects in DN by inhibiting fibrosis and reducing inflammation via the JUN, IL-6, and TNF-signaling pathways. We studied the therapeutic effects of DSS on db/db mice *in vivo* to further support this hypothesis.

We explored the curative effects of DSS in db/db mice with the goal of providing further support for this hypothesis. The *in vivo* data showed that DSS decreased Scr levels and considerably reduced pathological damage in db/db mice. DSS substantially decreased JNK, P-JNK, c-Jun, and P-c-Jun expression *in vivo*, according to the western blotting. We also found that DSS reduced the expression of ICAM-1, IL-6, and TNF-*α*; this finding was validated by H&E staining, which revealed inflammatory infiltration. Inflammation is the initial reaction of the body to kidney damage. JNK is a mitogen-activated protein kinase subfamily protein that regulates critical biological functions, such as cell proliferation, differentiation, and apoptosis [32]. The JNK pathway is activated by a variety of stimuli associated with kidney damage, including proinflammatory cytokines, danger-associated molecular pattern ligands (alarmins), oxidative stress, profibrotic agents, and nephrotoxins [33]. Seen in the most types of human diabetic nephropathy, the activation of the JNK pathway can worsen symptoms and inhibiting the JNK pathway is a therapeutic target for diabetic nephropathy [34]. JNKs not only have a role in the synthesis of a variety of inflammatory cytokines but also the expression of proinflammatory cytokines, such as TNF-*α* and IL-6, is linked to the continued activation of JNK1 [35, 36]. Activated by multiple pathogens and other inflammatory diseases, JNK is able to phosphorylate c-Jun, triggering a series of phosphorylation cascade events and regulating critical downstream effector molecules such as TNF-*α*, IL-6, IL-1, ICAM-1 to exert its biological impact [37–39]. Intercellular adhesion molecule 1 (ICAM-1) induces a proinflammatory and proatherogenic response in atherosis, insulin resistance, and the development of coronary disease [40]. Recently, clinical studies have revealed that increased serum/plasma ICAM-1 levels are significantly connected with albuminuria in T1D and T2D patients [41, 42]. As a pleiotropic cytokine, IL-6 signaling is critical in the regulation of inflammation and immunological responses [43]. Numerous studies have shown that IL-6 is essential to the etiology and progression of DN via gp130–STAT3 dependent processes and also acts locally in tissue remodeling and immune cell infiltration [44]. TNF-*α* mediates inflammatory processes and is also implicated in the progression of diabetic complications [45]. Almost all that reside in the kidney can generate TNF-*α* [46]. There is growing evidence that elevated TNF-*α* concentrations enhance sodium absorption, resulting in renal hypertrophy and salt retention, which are characteristic abnormalities during the early stages of diabetic neuropathy [47]. We demonstrated that DSS may suppress inflammatory and fibrotic responses in db/db mice through network pharmacology analysis.

## 7. Conclusion

The active pharmacological components and mechanisms by which DSS suppresses inflammation in DN were demonstrated using an integrated approach, including network pharmacology and experimental validation. We found that DSS suppresses inflammation primarily by regulating the JNK signaling pathway. In conclusion, the combination of network pharmacology and experimental validation was useful in defining the mechanism of the action of DSS.

## Figures and Tables

**Table 1:** Information for candidate active components from DSS.

Mol ID	Molecule name	OB (%)	DL
MOL000358	Beta-sitosterol	36.91	0.75
MOL000449	Stigmasterol	43.83	0.76
MOL001910	11alpha,12alpha-epoxy-3beta-23-dihydroxy-30-norolean-20-en-28,12beta-olide	64.77	0.38
MOL001918	Paeoniflorgenone	87.59	0.37
MOL001919	(3S,5R,8R,9R,10S,14S)-3,17-dihydroxy-4,4,8,10,14-pentamethyl-2,3,5,6,7,9-hexahydro-1H-cyclopenta[a]phenanthrene-15,16-dione	43.56	0.53
MOL001921	Lactiflorin	49.12	0.8
MOL001924	Paeoniflorin	53.87	0.79
MOL001925	Paeoniflorin_qt	68.18	0.4
MOL001928	Albiflorin_qt	66.64	0.33
MOL001930	Benzoyl paeoniflorin	31.27	0.75
MOL000211	Mairin	55.38	0.78
MOL000359	Sitosterol	36.91	0.75
MOL000422	Kaempferol	41.88	0.24
MOL000492	(+)-catechin	54.83	0.24
MOL000273	(2R)-2-[(3S,5R,10S,13R,14R,16R,17R)-3,16-dihydroxy-4,4,10,13,14-pentamethyl-2,3,5,6,12,15,16,17-octahydro-1H-cyclopenta[a]phenanthren-17-yl]-6-methylhept-5-enoic acid	30.93	0.81
MOL000275	Trametenolic acid	38.71	0.8
MOL000276	7,9(11)-dehydropachymic acid	35.11	0.81
MOL000279	Cerevisterol	37.96	0.77
MOL000280	(2R)-2-[(3S,5R,10S,13R,14R,16R,17R)-3,16-dihydroxy-4,4,10,13,14-pentamethyl-2,3,5,6,12,15,16,17-octahydro-1H-cyclopenta[a]phenanthren-17-yl]-5-isopropyl-hex-5-enoic acid	31.07	0.82
MOL000282	Ergosta-7,22E-dien-3beta-ol	43.51	0.72
MOL000283	Ergosterol peroxide	40.36	0.81
MOL000285	(2R)-2-[(5R,10S,13R,14R,16R,17R)-16-hydroxy-3-keto-4,4,10,13,14-pentamethyl-1,2,5,6,12,15,16,17-octahydrocyclopenta[a]phenanthren-17-yl]-5-isopropyl-hex-5-enoic acid	38.26	0.82
MOL000287	3beta-Hydroxy-24-methylene-8-lanostene-21-oic acid	38.7	0.81
MOL000289	Pachymic acid	33.63	0.81
MOL000290	Poricoic acid A	30.61	0.76
MOL000291	Poricoic acid B	30.52	0.75
MOL000292	Poricoic acid C	38.15	0.75
MOL000296	Hederagenin	36.91	0.75
MOL000300	Dehydroeburicoic acid	44.17	0.83
MOL000072	8-ethoxyatractylenolide-276.41	1.08	0
MOL000033	(3S,8S,9S,10R,13R,14S,17R)-10,13-dimethyl-17-[(2R,5S)-5-propan-2-yloctan-2-yl]-2,3,4,7,8,9,11,12,14.15,16,17-dodecahydro-1H-cyclopenta[a]phenanthren-3-ol	36.23	0.78
MOL000028	Amyrin	39.51	0.76
MOL000049	3-acetoxyatractylone	54.07	0.22
MOL000021	14-acetyl-12-senecioyl-2E,8E,10E-atractylentriol	60.31	0.31
MOL000020	12-senecioyl-2E,8E,10E-atractylentriol	62.4	0.22
MOL000022	14-acetyl-12-senecioyl-2E,8Z,10E-atractylentriol	63.37	0.3
MOL000830	Alisol B	34.47	0.82
MOL000831	Alisol B monoacetate	35.58	0.81
MOL000832	Alisol,b,23-acetate	32.52	0.82
MOL000849	16-methoxyalisol B monoacetate	32.43	0.77
MOL000853	Alisol B	36.76	0.82
MOL000854	Alisol C	32.7	0.82
MOL000856	Alisol C monoacetate	33.06	0.83
MOL002464	1-monolinolein	37.18	0.3
MOL000862	[(1S,3R)-1-[(2R)-3,3-dimethyloxiran-2-yl]-3-[(5R,8S,9S,10S,11S,14R)-11-hydroxy-4,4,8,10,14-pentamethyl-3-oxo-1,2,5,6,7,9,11,12,15,16-decahydrocyclopenta[a]phenanthren-17-yl]butyl] acetate	35.58	0.81
MOL001494	Mandenol	42	0.19
MOL002135	Myricanone	40.6	0.51
MOL002140	Perlolyrine	65.95	0.27
MOL002151	Senkyunone	47.66	0.24
MOL002157	Wallichilide	42.31	0.71
MOL000433	FA	68.96	0.71

## Data Availability

The datasets used and/or analyzed during the current study are available from the corresponding author upon reasonable request.
